# Fast growth of large-grain and continuous MoS_2_ films through a self-capping vapor-liquid-solid method

**DOI:** 10.1038/s41467-020-17517-6

**Published:** 2020-07-23

**Authors:** Ming-Chiang Chang, Po-Hsun Ho, Mao-Feng Tseng, Fang-Yuan Lin, Cheng-Hung Hou, I-Kuan Lin, Hsin Wang, Pin-Pin Huang, Chun-Hao Chiang, Yueh-Chiang Yang, I-Ta Wang, He-Yun Du, Cheng-Yen Wen, Jing-Jong Shyue, Chun-Wei Chen, Kuei-Hsien Chen, Po-Wen Chiu, Li-Chyong Chen

**Affiliations:** 1 Institute of Atomic and Molecular Sciences, Academia Sinica, Taipei, 10617 Taiwan; 2 Department of Electrical Engineering, National Tsing Hua University, Hsinchu, 30013 Taiwan; 3 Center of Atomic Initiative for New Materials, National Taiwan University, Taipei, 106 Taiwan; 4 Department of Electrical Engineering, Stanford University, Stanford, CA 94305 USA; 5 Department of Chemistry, National Taiwan Normal University, Taipei, 116 Taiwan; 6 Research Center for Applied Sciences, Academia Sinica, Taipei, 11529 Taiwan; 7 Department of Materials Science and Engineering, National Taiwan University, Taipei, 106 Taiwan; 8 Center for Condensed Matter Sciences, National Taiwan University, Taipei, 10617 Taiwan

**Keywords:** Engineering, Materials science, Nanoscience and technology

## Abstract

Most chemical vapor deposition methods for transition metal dichalcogenides use an extremely small amount of precursor to render large single-crystal flakes, which usually causes low coverage of the materials on the substrate. In this study, a self-capping vapor-liquid-solid reaction is proposed to fabricate large-grain, continuous MoS_2_ films. An intermediate liquid phase-Na_2_Mo_2_O_7_ is formed through a eutectic reaction of MoO_3_ and NaF, followed by being sulfurized into MoS_2_. The as-formed MoS_2_ seeds function as a capping layer that reduces the nucleation density and promotes lateral growth. By tuning the driving force of the reaction, large mono/bilayer (1.1 mm/200 μm) flakes or full-coverage films (with a record-high average grain size of 450 μm) can be grown on centimeter-scale substrates. The field-effect transistors fabricated from the full-coverage films show high mobility (33 and 49 cm^2^ V^−1^ s^−1^ for the mono and bilayer regions) and on/off ratio (1 ~ 5 × 10^8^) across a 1.5 cm × 1.5 cm region.

## Abstract (web summary)

Here, the authors develop a self-capping vapour-liquid-solid reaction to fabricate large-grain continuous MoS_2_ films, whereby an intermediate liquid phase-Na_2_Mo_2_O_7_ is formed through a eutectic reaction of MoO_3_ and NaF, followed by sulphurisation into MoS_2_.

## Introduction

Apart from graphene, transition metal dichalcogenides (TMDs) with atomic thickness are the most renowned two-dimensional (2D) materials because of their excellent electrical and optical properties^1–6^. Their robust physical properties in atmosphere enable their practical applications in novel optoelectronic devices^7,8^. For electronics, TMDs with atomic thickness, which inherently have no surface dangling bonds, are immune to mobility degradation and short channel effects in contrast to conventional three-dimensional materials, such as Si and GaAs^9–11^. Such materials have layer-dependent bandgaps from near infrared to visible regions^12,13^, and thus, TMDs are favorable for energy or optical applications^7,8,14,15^. Furthermore, the difference between Berry curvature at the K and K′ valleys of monolayer TMDs generates new opportunities for valleytronics^16,17^. Despite these remarkable properties, TMDs still have limitations arising from spatial nonuniformity. Therefore, the fabrication of high-quality and large-grain films is thus crucial for TMDs. Currently, chemical vapor deposition (CVD) is the most recognized method for producing high-quality monolayer TMDs because of its low cost and scalability^18–22^. Conventional CVD methods of TMD fabrication are based on the reaction of gas-phase chalcogens (e.g., S and Se) and metal oxides (e.g., MoO_3_ and WO_3_)^18–22^. Generally, in gas-phase reactions, the grain size of TMDs is limited by the high nucleation density and typically is <500 µm. Recently, researchers have used various methods, which include fabricating at a high temperature^23^, inserting diffusion barriers^24^, and using an extremely small amount of precursors^25^, to reduce nucleation density and to increase the surface diffusion length for growing large TMD crystals. These methods can produce comparatively large crystals but considerably reduce the coverage of TMD crystals^25^, which hinders their practical applications.

For bulk materials, Czochralski^26,27^ method enables the production of a single-crystal ingot with a diameter of up to 300 mm by vertically pulling a solid seed crystal from a liquid source^28^. This method provides the unprecedentedly high uniformity of conventional bulk materials at an ultra-large scale. Moreover, a liquid source can more easily dissolve other solid dopants than a gas–gas reaction^29^. Therefore, it is desirable to grow solid crystals from liquid sources. For the growth of TMDs, Li et al. recently proposes the vapor–liquid–solid (VLS) reaction for fabricating high-quality MoS_2_ nanoribbons from a liquid precursor on a sodium chloride (NaCl) single crystal^30^. First, NaCl reacts with MoO_3_ to form a eutectic compound (Na_2_Mo_2_O_7_), which has a relatively lower melting point and exists in a liquid phase under growth conditions (generally 700–800 °C). Second, the sulfur vapor is rapidly dissolved into the liquid and reacts to form a solid-state monolayer MoS_2_ on NaCl. However, because of the low wettability between the NaCl and liquid Na_2_Mo_2_O_7_ droplets, this method can only generate MoS_2_ nanoribbons, which considerably limits its application. This problem can be solved by growing on other substrates with a better wettability^31^.

Herein, a self-capping vapor–liquid–solid (SCVLS) reaction, which can grow large single crystals and full-coverage TMD films, is proposed. A solid precursor comprising ultra-thin MoO_3_, SiO_2_, and NaF layers was used for the controllable eutectic reaction of MoO_3_ and NaF at high temperature. The as-formed eutectic liquid (Na_2_Mo_2_O_7_) rose to the surface and was sulfurized into MoS_2_ seeds. These seeds, acted as a self-capping layer, redirected the rising liquid into a horizontal direction. The residual liquid was continuously pushed along the growth direction and eventually sulfurized to form new MoS_2_ at the edge of the MoS_2_ seeds. This growth mechanism enables fabrication of ultra-large (~1.1 mm) single crystals. Moreover, continuous large-area MoS_2_ film with large-grain size (~450 µm) can also be fabricated using thicker precursor. By controlling the kinetic factors of this reaction, the layer number can be controlled and large bilayer MoS_2_ (~200 µm) can be achieved. In this study, the quality and uniformity of MoS_2_ grown using this method are evaluated through electron microscopy, optical spectroscopy, and electrical measurements. For electrical measurements, both mono- and bilayer MoS_2_ field-effect transistors (FETs) show high mobility (33 and 49 cm^2^V^−1^ s^−1^), large on/off ratio (5 × 10^8^), and high current density (up to 230 and 390 µA µm^−1^). The large-grain, continuous film exhibits high performance across a 1.5 ×1.5 cm area, making the SCVLS method promising for practical applications.

## Result

### Material synthesis and growth mechanism

**Fig. 1:**
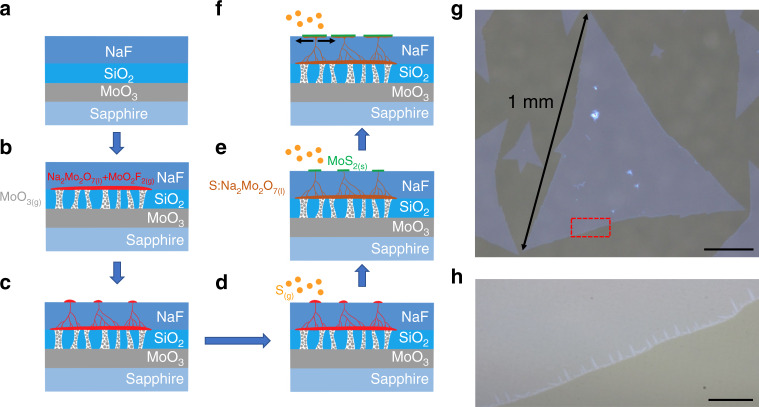
Schematics of SCVLS growth mechanism and the grown MoS_2_. **a** Structure of the solid precursor used for the SCVLS method. **b** At growth temperature, MoO_3_ vaporized and penetrated through the SiO_2_ diffusion membrane. MoO_3_ and NaF reacted to form liquid-phase Na_2_Mo_2_O_7_ (colored in red) at the growth temperature. **c** Through reactive digging and the capillary effect, the liquid precursor gradually rose to the NaF matrix surface. **d** Sulfur vapor was introduced into the system and started to dissolve into the Na_2_Mo_2_O_7_ liquid. **e** Liquid precursor sulfurized into the MoS_2_ seed layer. **f** Capped by the MoS_2_, the emerging liquid was redirected horizontally and converted into MoS_2_ when it contacted and dissolved sulfur vapor at the edge of the MoS_2_ flakes. **g** A 1-mm MoS_2_ flake grown through the SCVLS method. Scale bar is 200 µm. **h** Magnified image of the MoS_2_ grain edge. The fringes at the edge indicate the presence of the liquid precursor during the growth process. Scale bar is 20 µm.

Figures 1a and S1 show that a smooth MoO_3_ layer was grown on c-plane sapphire through plasma-enhanced atomic layer deposition (PEALD). SiO_2_ and NaF layers were stacked on top of the MoO_3_ layer through sputtering and thermal evaporation, respectively. The SiO_2_ layer acted as a diffusion membrane to control the amount of MoO_3_ vapor that broke the SiO_2_ layer (Fig. 1b and Supplementary Fig. 2), diffused upward and reacted with the NaF layer at a temperature higher than 500 °C to form liquid-phase Na_2_Mo_2_O_7_ and gas-phase MoO_2_F_2_ (Fig. 1b and Supplementary Fig. 3). Simultaneously, the consumption of NaF generated holes and pathways in the NaF layer, which allowed Na_2_Mo_2_O_7_ and MoO_2_F_2_ to gradually rise to the top surface of the NaF through the pressure gradient and capillary phenomenon (Fig. 1c). Meanwhile, sulfur vapor was introduced into the system and rapidly dissolved in the eutectic liquid (Na_2_Mo_2_O_7_) that rose to the surface. As discussed in the first VLS paper, the products of this VLS reaction were MoS_2(s)_ and sulfur oxides (SO_2(g)_ and SO_3(g)_)^30^. Moreover, the molten liquid surface provided a temporarily atomic-flat and defection-free surface with a low nucleation density^23,32^. The oversaturated MoS_2_ precipitated as seed layers on the liquid surface (Fig. 1e). The as-formed MoS_2_ seed layers blocked the route for sulfur to dissolve into the liquid and redirected the underlying liquid to move horizontally. The unsaturated liquid then emerged to the surface at the MoS_2_ edge, and this was where the SCVLS reaction primarily occurred. Therefore, MoS_2_ laterally grew into large crystals (Fig. 1f, g). Millimeter-sized MoS_2_ single crystals were obtained using this SCVLS method. The large triangular MoS_2_ flakes are single crystals in nature, as validated by diffraction analysis at multiple spots in a large flake (Supplementary Fig. 4). The zoom-in image of a MoS_2_ edge exhibits many bilayer fringes (Fig. 1h), which were a result of the precipitation of the residual liquid at the edges of the MoS_2_ flakes during the rapid cooling process. These fringes validate the existence of the liquid phase during growth and the aforementioned mechanism. In contrast to the conventional CVD method, wherein the nonuniform gas flow in the furnace often gives resultant film of poorer uniformity^33^, extending the SCVLS method to a wafer scale is facile because the precursor rises uniformly from the bottom surface of the growth substrate. Supplementary Fig. 5 shows a full-coverage MoS_2_ film grown on a sapphire of 3 × 3 cm^2^ (size was only limited by the CVD tube size). This process can also be extended to grow MoSe_2_ by replacing sulfur with selenium, result of which is shown in Supplementary Fig. 6.

### Characterization of as-grown material

**Fig. 2:**
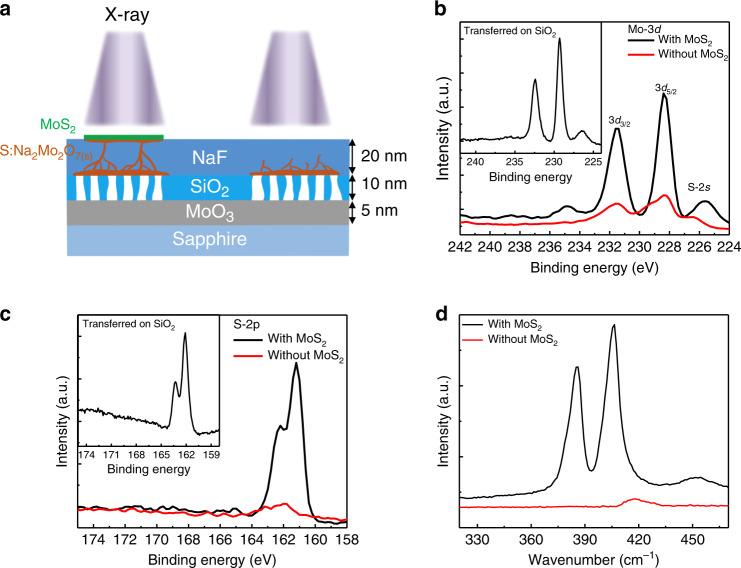
Characterization of the MoS_2_ monolayer and remaining solid precursors. **a** Schematic of the sampling areas of XPS. XPS data of **b** Mo-3*d* and **c** S-2*p* on sites that were covered (black) and not covered (red) with MoS_2_. The broad peak of Mo-3*d* at sites that were not covered with MoS_2_ indicates the complex chemical environment of Mo below the surface. Insets in **b** and **c** are the Mo-3*d* and S-2*p* of the MoS_2_ film transferred onto a SiO_2_ substrate. **d** Raman spectra taken form positions with (black) and without (red) MoS_2_ coverage.

Compared with the conventional CVD technique, the final products of this SCVLS method were more complex (Fig. 2a), which comprised two parts: the top MoS_2_ layer and the complex solid products generated by the quenched liquid within the NaF matrix. During the sulfurization process, sulfur vapor was primarily dissolved into the liquid through the exposed liquid–gas interface, and thus, the oversaturated liquid could continuously precipitate MoS_2_ at the edge of the MoS_2_ seeds. However, the reactions were not limited to the top surface. With a lower sulfur concentration, some incomplete sulfurization reactions and precipitations were observed within the NaF matrix (Supplementary Fig. 7). Upon cooling, the unsaturated liquid solidified and resided below the MoS_2_ flakes or was buried in the NaF matrix (Fig. 2a). X-ray photoelectron spectroscopy (XPS) was used to analyze the final products. Figure 2b, c shows Mo-3*d* and S-2*p* spectra obtained from regions covered by large MoS_2_ flakes and regions with the exposed NaF matrix, respectively. For a region covered with MoS_2_, the Mo-3*d* peak of ~230–228 eV could be deconvoluted into sharp Mo^4+^ and broad Mo^*x*+^. The Mo^4+^ signal was obtained from the top MoS_2_, and the Mo^*x*+^ signal was obtained from the precipitates and solidified liquid phase in the NaF matrix, as shown using an XPS depth profile (Supplementary Fig. 7). With a lower sulfur concentration in the matrix, the possible products for the precipitate and quenched liquid were amorphous MoS_2_, MoO_2_, and MoS_*x*_O_*y*_, comprising the Mo^*x*+^ signal. In addition, a small peak at ~235 eV indicates Mo^6+^, which is the peak from Na_2_Mo_2_O_7_, indicating the presence of residual unreacted precursor below MoS_2_; this supports the as-proposed horizontal transport of the liquid. For a region without MoS_2_, only a small amount of sulfur diffused into the NaF matrix and reacted with the liquid below the surface. Figure 2b, c shows a considerably weaker Mo^*x*+^ and sulfur signal, which indicates that no liquid rose to the top surface. The resultant products were further characterized using Raman spectroscopy (Fig. 2d). The region covered with MoS_2_ exhibited sharp E_2g_ and A_1g_ peaks with a spacing of 19.5 cm^−1^, thus validating the high quality and monolayer characteristics for the as-grown MoS_2_. The region that was not covered by MoS_2_ exhibited no significant Raman signal, which validated the absence of any crystalline product in the NaF matrix. The as-grown monolayer MoS_2_ could be readily transferred to various substrates using the conventional polymethyl-methacrylate (PMMA) method. Atomic force microscopy images and photoluminescence spectrum in Supplementary Fig. 8 and 9 also confirm the monolayer property. The insets of Fig. 2b, c show the XPS results of the as-transferred MoS_2_ on silica. Compared with the as-grown sample, the transferred MoS_2_ exhibited sharp and clean Mo^4+^ signals at 229.3 and 232.4 eV. The Mo–S ratio, which was calculated by integrating the area of the Mo and S signals, was 1:2, as expected for high-quality MoS_2_.

### Controlling the coverage and thickness of MoS_2_

**Fig. 3:**
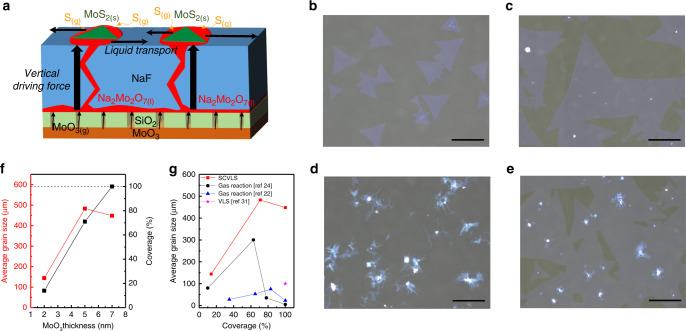
Driving forces of SCVLS reaction. **a** Schematic of the vertical driving force and horizontal liquid transport. Optical microscopy images of MoS_2_ grown using **b** 2-nm **c** 5-nm, and **d** 7-nm MoO_3_ as a solid precursor. The growth time was 10 min. **e** 1-min growth using the same precursor sample as in **d**. The scale bars in **b**, **d**, and **e** are 200 µm and the one in **c** is 300 µm. **f** Average grain size and coverage of the MoS_2_ flakes as a function of MoO_3_ precursor thickness. **g** Comparison of SCVLS, VLS, and gas-phase CVD. SCVLS reaction enables a relatively large-grain size when a full-coverage film was achieved.

With a suitable sulfur source, the growth rate of SCVLS process was controlled by horizontal transport rate of the liquid. Rapid horizontal mass transport of the liquid was crucial for growing large monolayer MoS_2_. The driving force stemmed from the diffusion and capillary phenomena of the high-pressure liquid and gas produced by the eutectic reaction (Fig. 3a). Because MoS_2_ layers covered the top surface and confined the liquid flow, the vertical driving force was redirected horizontally, and both vertical and horizontal liquid transport was promoted with increasing amount of liquid source. The rapid SCVLS reaction could thus be performed under this condition, and the rapid growth rate abruptly increased the grain size of MoS_2_. However, when the driving force was weak, the low growth rate would result in more nucleation seeds on NaF, thus reducing the average size of the MoS_2_ crystals. In some regions, the weak driving force was not sufficient to push the liquid to the surface. The sulfur vapor would slowly diffuse into the NaF matrix, react with the liquid, and eventually solidify. Therefore, no MoS_2_ was grown on the surface under this condition, and this phenomenon reduced the coverage of MoS_2_. Here, the vertical driving force was controlled using different amounts of MoO_3_ sources. Figure 3b–d shows the optical images of the as-grown MoS_2_ with different thicknesses of MoO_3_ precursor layers. The grain size and coverage of MoS_2_ abruptly increased with the increasing thickness of MoO_3_ precursor (Fig. 3f). In order to estimate the grain size of the full-coverage film (Fig. 3d), the growth time was reduced from 10 (Fig. 3d) to 1 min (Fig. 3e), to monitor the grain size before grains merged into continuous film. The average grain size of the full-coverage film was ~450 µm, which is the largest recorded average grain size for completely covered MoS_2_ film. Although there are some thick islands on the film (Fig. 3d and Supplementary Fig. 10), this large-grain continuous film can still demonstrate outstanding electrical performance shown in the later section. The trend of the coverage and average grain size versus the precursor thickness in SCVLS (Fig. 3f) is very different from that for the common gas-phase reaction CVD. For gas-phase CVD, the average grain size of the continuous film is reduced by a factor of 10–100 compared with the largest isolated crystals because the larger amount of the precursor for growing continuous film abruptly led to the increased nucleation density and thus reduced grain size (Fig. 3g)^25,34^. However, for SCVLS method, the average grain sizes of the continuous film (450 µm) and largest isolated crystals (500 µm) are similar because of the self-capping effect and the fast transport of liquid. Furthermore, coverage of ~82% was reached within 1 min of fabrication (Fig. 3e), which demonstrates the rapid growth rate (370 µm/min, see Supplementary Fig. 11). This may be a result from the fast transport of liquid assisted by the fluoride surface, which has been shown to enhance growth rate of 2D materials^35^. The driving force and nucleation density could be further controlled by tuning the thickness of SiO_2_ membranes, growth temperature, and the thickness of NaF (Supplementary Figs. 12–14).

**Fig. 4:**
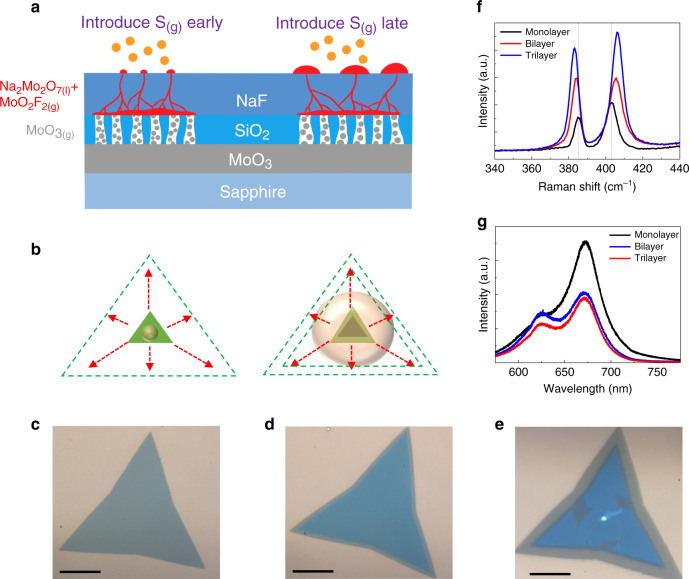
Dynamic effect on SCVLS method. **a** Schematic of the timing of introducing sulfur vapor affecting the final growth product. **b** When sulfur vapor was introduced early, the Na_2_Mo_2_O_7_ precursor rapidly formed the MoS_2_ seed layer when exposed on the surface (left). When sulfur vapor was introduced later, the MoS_2_ seed layer was formed at the solid–liquid interface, thus leaving a droplet of the liquid precursor on top of the interface. This droplet was later sulfurized into the second layer of MoS_2_ (right). Optical images of the transferred **c** monolayer, **d** bilayer, and **e** trilayer MoS_2_. Scale bars are 50 µm. **f** Raman and **g** photoluminescence spectra of the monolayer, bilayer, and trilayer MoS_2_.

In addition to large-grain continuous film, layer-controlled growth of bilayer and multilayer are also attractive because of the better electrical performance of few-layer MoS_2_^36–39^. SCVLS can also control the layer number of MoS_2_ with its special growth mechanism we proposed. In the growth condition of previous paragraphs, sulfur vapor was introduced before or during the liquid’s rise to the surface. The small liquid droplets rapidly dissolved sulfur and formed monolayer MoS_2_ capping seeds, which promoted the horizontal mass transport and formed large monolayer MoS_2_ grains. It is noteworthy to mention that the SCVLS reaction can be dynamically controlled by changing the timing of sulfurization (Fig. 4a). When sulfur vapor was introduced later, the emerged liquid would form into a large droplet (Supplementary Fig. 15). During sulfurization, the as-formed small MoS_2_ seeds were buried in the oversaturated liquid. Under this condition, fresh MoS_2_ could be formed at the edge of the original seeds, and a second layer could be grown on the MoS_2_ seeds (Fig. 4b). Large bilayer MoS_2_ crystals could be fabricated by delaying the sulfurization timing for 2 min. Trilayer MoS_2_ was occasionally observed when sulfurization is delayed. Figure 4c–e are the optical images of the transferred mono-, bi-, and trilayer MoS_2_ on SiO_2_/Si substrates, respectively. The clear optical contrast shows the characteristics of the mono-, bi-, and trilayers of each MoS_2_. AFM images in Supplementary Fig. 16 also confirm the thickness of these samples. Figure 4d, e shows that the second and third layers are well-aligned with the bottom MoS_2_ layer, thus indicating the epitaxial growth of an excess MoS_2_ layer. The diffraction patterns manifest a 2H-type stacking order of the SCVLS reaction (Supplementary Fig. 17)^30^. Raman spectra in Fig. 4f further validate the layer number and strong interaction between the mono-, bi-, and trilayer MoS_2_ grown using the SCVLS method. The peak separations of E_2g_ and A_1g_ are 18.0, 21.5, and 23.2 cm^−1^, which are similar to the values previously reported for exfoliated MoS_2_^40^. The strong interaction between each layer changes the dielectric environment, thus softening the in-plane E_2g_ mode (red-shift). Moreover, the strong interaction between interlayer S increases the restoring force, thus stiffening the out-of-plane A_1g_ mode (blue shift)^40^. The photoluminescence spectra in Fig. 4g exhibit clear quenched signals for bilayer and trilayer MoS_2_ because of the direct–indirect band gap transition for monolayer and bilayer MoS_2_^4,6^. By employing dynamic control of sulfurization, a large bilayer single crystal (200 µm) is successfully synthesized, the grain size of which is comparable to the large bilayer crystals in the previous studies^37,38^. Moreover, the ability to change the layer number of MoS_2_ by controlling the size of the droplet validated the SCVLS mechanism proposed in Fig. 1.

### High-performance FET device

**Fig. 5:**
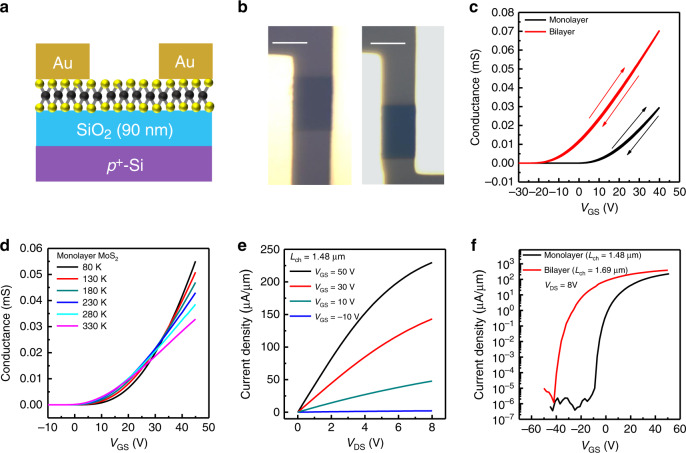
Transport properties of the MoS_2_ grown through the SCVLS method. **a** Schematic image of a back-gate monolayer MoS_2_ transistor. **b** Optical image of a monolayer and bilayer FETs. Both scale bars are 5 µm. **c** Gate-dependent conductance of devices shown in **b**. **d** Temperature-dependent transport property of the monolayer MoS_2_ FET shown in **b**. A clear MIT is observed at *V*_*GS*_ of 30 V. **e** *V*_*DS*_-dependent source-drain current density of the monolayer device at different gate voltages. The channel length is 1.48 μm. **f** Log plot of the gate-dependent current density of the short channel mono- and bilayer devices under an 8-V source-drain bias. The on/off ratio is 5 × 10^8^.

**Fig. 6:**
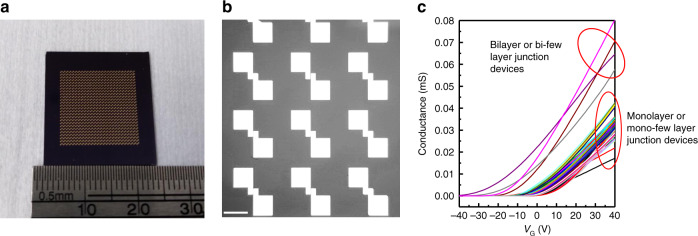
Transport properties of the large-grain, continuous film. **a** Photo-image of the as-fabricated FETs across a 1.5 × 1.5-cm region. **b** Optical image of FET devices. The scale bar is 150 μm. The fine feature is shown in Supplementary Fig. 18. **c** Gate-dependent conductance of the devices across the large area.

The electrical properties of the large monolayer MoS_2_ crystal grown by the SCVLS method was examined by measuring the transport properties of MoS_2_ FETs. Figure 5a shows an FET with a back-gate structure and 90-nm-thick SiO_2_. Both mono- and bilayer MoS_2_ FETs were fabricated as shown in Fig. 5b and Supplementary Fig. 18. Figure 5c displays the gate-dependent conductance of the MoS_2_ FETs. Both devices have a very small hysteresis, indicating low defect and impurity induced trap density in the SCVLS growth and the device fabrication processes. The field-effect mobility was calculated using $\mu _{FE} = \frac{{L_{CH}}}{W}\frac{1}{{C_G}}\frac{{dG}}{{dV_{GS}}}$, where *C*_*G*_, *L*_*CH*_, *W*, V_*GS*_, and *G* stand for the back-gate capacitance, channel length, channel width, back-gate voltage, and sheet conductance of the channel, respectively. Because of its higher carrier density and stronger charge screening effect, bilayer MoS_2_ has a smaller threshold voltage (*V*_*th*_) and higher mobility. The mobilities of the mono- and bilayer MoS_2_ FETs are 33 and 49 cm^2^ V^−1^ S^−1^, respectively. These values are comparable to the exfoliated MoS_2_^41^ and the best reported values of CVD MoS_2_ on SiO_2_^38,42,43^, showing the high quality of MoS_2_ grown through the SCVLS method. The temperature-dependent transport also confirms the quality of SCVLS MoS_2_ as shown in Fig. 5d. For the monolayer device, a clear metal–insulator transition (MIT) was observed, which is generally detected when using a high-*k* dielectric layer to reduce Columbic scattering in the MoS_2_ channel^2^. For back-gate devices without a high-*k* dielectric layer, a clear MIT occurs only when using high-quality MoS_2_ with a low concentration of sulfur vacancies. In general, according to the Ioffe-Regel criterion^44^, MIT occurs when the critical channel conductance is approximately one quantum conductivity (*e*^2^/*h*)^2,44^. If the crossover point is in a lower carrier concentration region, this directly reflects the high-mobility property of MoS_2_^45,46^. The carrier concentration of the transition point is calculated using

1 $$n_{{\mathrm{MIT}}} = \frac{{C_G}}{e}\left( {V_T - V_{{\mathrm{MIT}}}} \right),$$

where *V*_*T*_ and *V*_MIT_ are the threshold voltage and voltage at which the MIT occurs, respectively^47,48^. The *n*_MIT_ of the SCVLS MoS_2_ in this study is 4.3 × 10^12^ cm^−2^, which is even lower than the previously reported intrinsic exfoliated MoS_2_ with low S-vacancy concentration^47^. This indicated the high quality and low sulfur vacancy concentration of the MoS_2_ fabricated using the SCVLS method. Figure 5e is the output characteristics of a short channel (1.48 μm, see Supplementary Fig. 19) monolayer FET at various back-gate voltages. The linear behavior in the low source-drain voltage (*V*_*DS*_) region shows a good Ohmic property of contacts. Figure 5f presents the semi-logarithmic of the gate-controlled current density. The device on/off ratio can reach 5 × 10^8^, with a subthreshold swing of 980 mV dec^−1^. The maximum current density in the monolayer MoS_2_ is 230 µA µm^−1^ (390 μA μm^−1^ for bilayer), potentially comparable to the optimal reported in consideration of the difference in contact geometry^38,42^. Table 1 lists the recently reported monolayer MoS_2_ back-gate FETs. MoS_2_ grown by the SCVLS method exhibits the largest grain size and remarkable electrical performance compared with other CVD techniques. The good uniformity of the large monolayer crystal is confirmed by measuring 18 FETs in a 1-mm crystal (Supplementary Fig. 20). In addition, with the capability of growing large-grain and continuous film, we fabricated hundreds of devices across a 1.5 × 1.5-cm area, as shown in Fig. 6a, b. Figure 6c is the gate-dependent conductance of a hundred devices in the whole area. Ninety percent of devices have pure monolayer channel and show high mobilities (34 ± 7 cm^2^ V^−1^ s^−1^) with small variation of *V*_*th*_ (4.9 ± 2.3 V). Devices have larger variation in mobility and *V*_*th*_ if their channels are bilayer, few-layer, mono-few-layer junction, or monolayer with a small few-layer flake on top. However, the mobilities are still high for all of the devices because of the large-grain monolayer underneath (Supplementary Fig. 21). This demonstrates the advantage of using the SCVLS method for practical applications.

**Table 1:** Comparison of the different CVD methods.

Growth method	Maximum grain size (single crystal)	Mobility (cm^2^ V^−1^ S^−1^)	Maximum current density (μA μm^−1^)	On/off ratio	Reference
CVD MoS_2_ (MoO_3_ + S, substrate control)^49^	200 μm	25		10^7^	ACS Nano 2015, 9, 4611
CVD MoS_2_ (MoO_3_ + S, flow control)^50^	300 μm	30		10^6^	Adv. Sci. 2016, 3, 1500033
CVD MoS_2_ (MoCl_5_ + DMS, NaCl catalyst)^51^	50 μm	10.4		10^7^	Nanotechnology 2017, 28, 465103
CVD MoS_2_ (MoO_3_ + S, molten Na:glass)^32^	563 μm	24	123	10^9^	Appl. Phys. Lett. 2018, 113, 202103
CVD MoS_2_ (MoO_3_ + S with PTAS salt)^42^	200 μm	35	270	10^7^	Nano Lett. 2018, 18, 4516
CVD MoS_2_ (Mo foil + S, soda–lime glass)^52^	400 μm	11.4		10^6^	Nat. Commun. 2018, 9, 979
SCVLS MoS_2_	1.1 mm	33	230	5 × 10^8^	This work

All transport data were obtained from the back-gate, monolayer MoS_2_ FETs for comparison.

## Discussion

In summary, a new concept of growing high-quality single-crystal 2D materials from the liquid precursor was proposed using the SCVLS method. The rapid horizontal mass transport promotes the lateral growth of 2D materials and allows the growth of MoS_2_ flakes as large as 1.1 mm. The self-capping effect drastically reduces the nucleation density even under large amount of precursor and results in a wafer scale, 100% coverage MoS_2_ films with an average grain size of up to 450 µm. It overcomes the bottleneck of the conventional gas-phase CVD reaction, which is the trade-off between coverage and grain size. The quality and uniformity of the as-grown MoS_2_ were carefully evaluated through the electrical properties of MoS_2_ FETs across a large area. High-mobility MoS_2_ devices are demonstrated across a 1.5 × 1.5-cm area. Moreover, this method is capable of fabricating large bilayer MoS_2_ crystals by controlling the timing of sulfurization. More sophisticated sulfurization precursor such as H_2_S is expected to improve the layer number control or the uniformity of continuous films. Fabricating crystals by using the liquid–solid reaction, such as in doping and alloying, is one expected niche of this SCVLS method, which provides a new approach for synthesizing industrial-grade 2D materials for practical applications in 2D electronics.

## Methods

### Preparation of solid precursor

3 × 3-cm c-plane (0001) sapphire substrate was cleaned first by deionized water then sonicated in acetone and isopropyl alcohol for 20 and 5 min, respectively. MoO_3_ film with well-controlled thickness was grown on top of sapphire substrates with a homemade PEALD system using Mo(CO)_6_ as precursor and oxygen plasma as the oxidation reactant. For each deposition cycle, a Mo(CO)_6_ precursor pulse is provided into the chamber, then the excess precursor is purged away by argon, and finally oxygen plasma (up to 200 W) is used to oxidize the precursor and form uniform MoO_3_ film. Thermal evaporation can be used to replace the PEALD process for depositing MoO_3_ but will result in worse MoS_2_ morphology (Supplementary Fig. 22). SiO_2_ film was deposited on top of MoO_3_ layer by sputtering a 3-inch SiO_2_ target with Ar plasma at a power density of 0.6 W/cm^2^ in a radio-frequency magnetron sputtering system. NaF thin film was deposited onto the sample by heating NaF powder (Acros, 97%) loaded in a Mo boat in a high vacuum evaporator chamber (<5 × 10^−5^ torr). For sputtering and thermal evaporation film, film thickness was monitored by a quartz crystal microbalance and the deposition rate was maintained at 0.1 Ås^−1^. Samples were attached to a spinning sample holder to obtain high uniformity.

### Growth of MoS_2_

High temperature growth was carried out in a 2-inch quartz tube and the temperature profile of the growth was controlled by a three-zone furnace. Sulfur powder (Aldrich, 99.98%), which was placed in an alumina crucible, and precursor sample held by a 3 × 3-cm^2^ quartz plate were placed at the center of first and third hearing zone, respectively, as depicted in Supplementary Fig. 1c. A 5 sccm H_2_ and 50 sccm Ar mixed gas flow was used as carrier gas and the pressure within the quartz tube was controlled to be 30 torr. The temperature at the sample ramped up at a rate of 40 °C min^−1^ to 800 °C and was held for 10 min. Sulfur vapor was introduced by ramping up the temperature at the first zone at a rate of 15 °C min^−1^ and was held at the desired temperature during growth. After growth, the furnace was turned off and was fast-cooled using an industrial fan. The temperature ramping profile is shown in Supplementary Fig. 1d. For monolayer MoS_2_ growth, A and B setpoints are reached at the same time. For bilayer MoS_2_ growth, the B setpoint is reached 2 min later than the A setpoint.

### Device fabrication and characterization

*p*-type heavily doped silicon wafers with 90-nm thermal oxide layers were used for back-gate FET devices. MoS_2_ films/isolated crystals were transferred to wafers through a conventional PMMA method. Optical lithography and oxygen plasma were used to define the MoS_2_ strips. Then, the second lithography process defined the source-drain patterns. A 50-nm gold layer was thermally evaporated under high vacuum as the source-drain and back-gate electrodes. Before measuring electrical properties, FETs were annealed at 120 °C under a 10^−3^ torr vacuum for 10 h in a probe station (Lakeshore). Gate and source-drain voltage were applied by Kethley 6487 picometers. Raman and photoluminescence spectra were measured by a confocal system equipped with a 476-nm laser. XPS spectra were obtained using PHI VersaProbe system. Transmission electron analysis (Supplementary Figs. 4, 17, and 23) was performed in JEOL AEM 2010F and JEOL AEM 2100F, which was equipped with a probe-type corrector for the spherical aberration of the objective lens. Both systems were operated at 200 kV for the analysis.

## Supplementary information

Supplementary Information

Peer Review File

## Data Availability

The data that support the findings of this study are available from the corresponding author on reasonable request.
