# Supplementary material for: Fast growth of large-grain and continuous MoS2 films through a self-capping vapor-liquid-solid method
Source: Nat Commun. 2020 Jul 23;11:3682. doi: 10.1038/s41467-020-17517-6 (PMC7378841; doi:10.1038/s41467-020-17517-6)
Supplement: Supplementary file 1 — Supplementary Information [file 41467_2020_17517_MOESM1_ESM.pdf]

Supplementary Information

Fast Growth of Large-Grain and Continuous MoS_2 Films through a Self-Capping Vapor-Liquid-Solid Method

Chang *et al.*

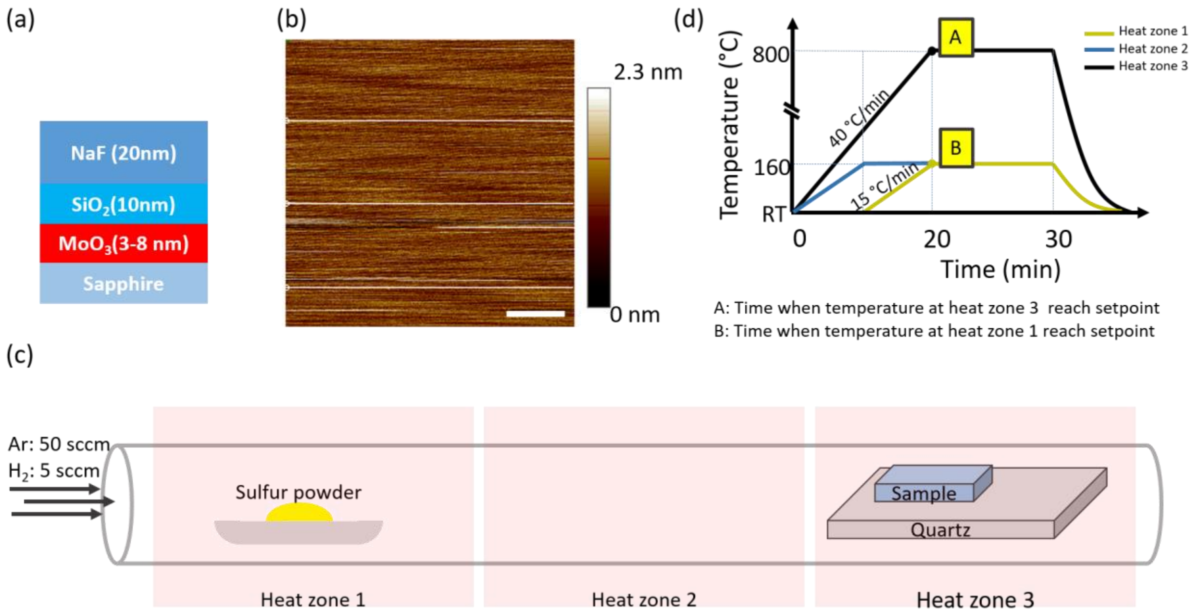

Supplementary Figure 1. SCVLS method. (a) Schematic cross section of precursor sample. (b) AFM image of the ALD grown MoO_3 on sapphire. The scale bar is 200 nm and the surface roughness is 0.25 nm (c) SCVLS growth setup. 0.3 g sulfur powder was put in an alumina boat and placed in the center of heat zone 1. Precursor samples were placed on top of the quartz plate and placed at the center of the heat zone 3. The distance between the sulfur source and precursor sample is about 39 cm. (d) Temperature ramping profile of the furnace. For growth of monolayer MoS_2, both sulfur and precursor reach the desired temperature at the same time. In other words, setpoint A and B were reached at the same time. For growth of bilayer MoS_2, setpoint B was reached 2 minutes later than A.

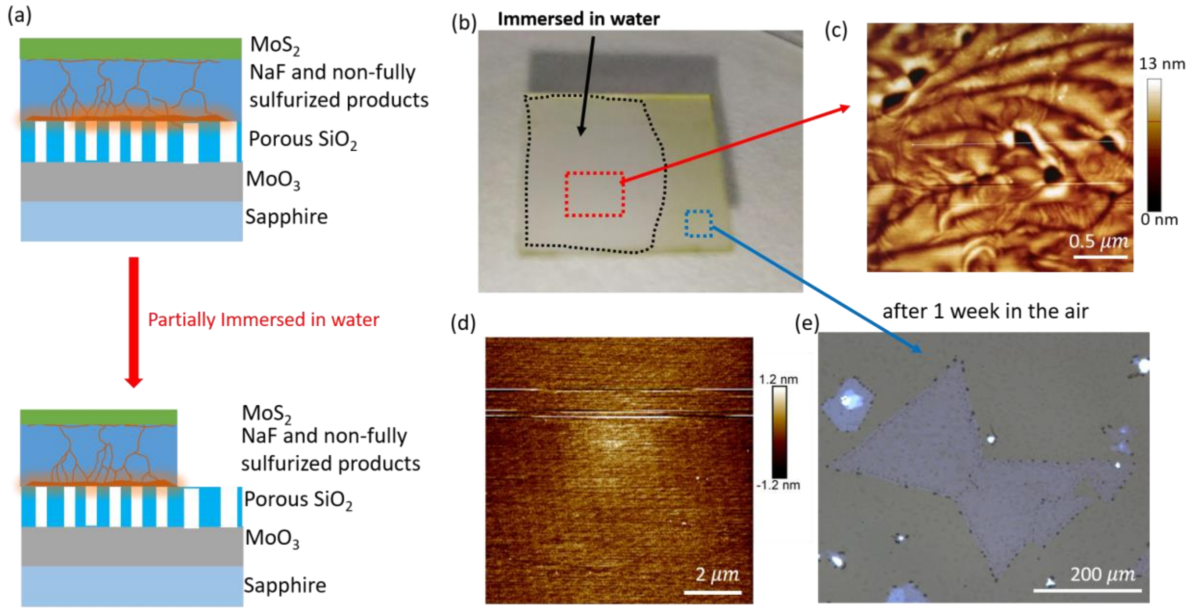

Supplementary Figure 2. Analysis of growth. In our SCVLS reaction, the NaF is used as a reagent and also a substrate for growing MoS_2, there should be excess NaF in the system. We use 20 nm NaF and 3-7 nm MoO_3 precursor for growing MoS_2 films. Considering the density and the molecule weight of NaF and MoO_3, the amount of NaF deposited is much more than the amount needed for the eutectic reaction. Therefore, there will be a NaF matrix with non-fully sulfurized products as shown in Figure 2(a) and (b), which are water soluble, after growth. They can be easily washed away by water (Supplementary Figure 2a and b), leaving a porous SiO_2 surface as shown in Figure S2c. This phenomenon, together with the depth profile XPS results, shows that the non-fully sulfurized residuals (MoO_x, MoS_xO_{2-x}) are buried in the water-soluble matrix, including NaF and $Na_2Mo_2O_7$. The porous SiO_2 also proves that the MoO_3 vapor breaks the original smooth SiO_2 layer (Supplementary Figure 2d) so that it can gradually react with NaF as in our proposed mechanism. In addition, the morphology of the sample changes (Supplementary Figure 2e) after 1 week because of the deliquescence. This proves that the MoS_2 is grown on a NaF matrix with non-fully sulfurized products and confirms our mechanism.

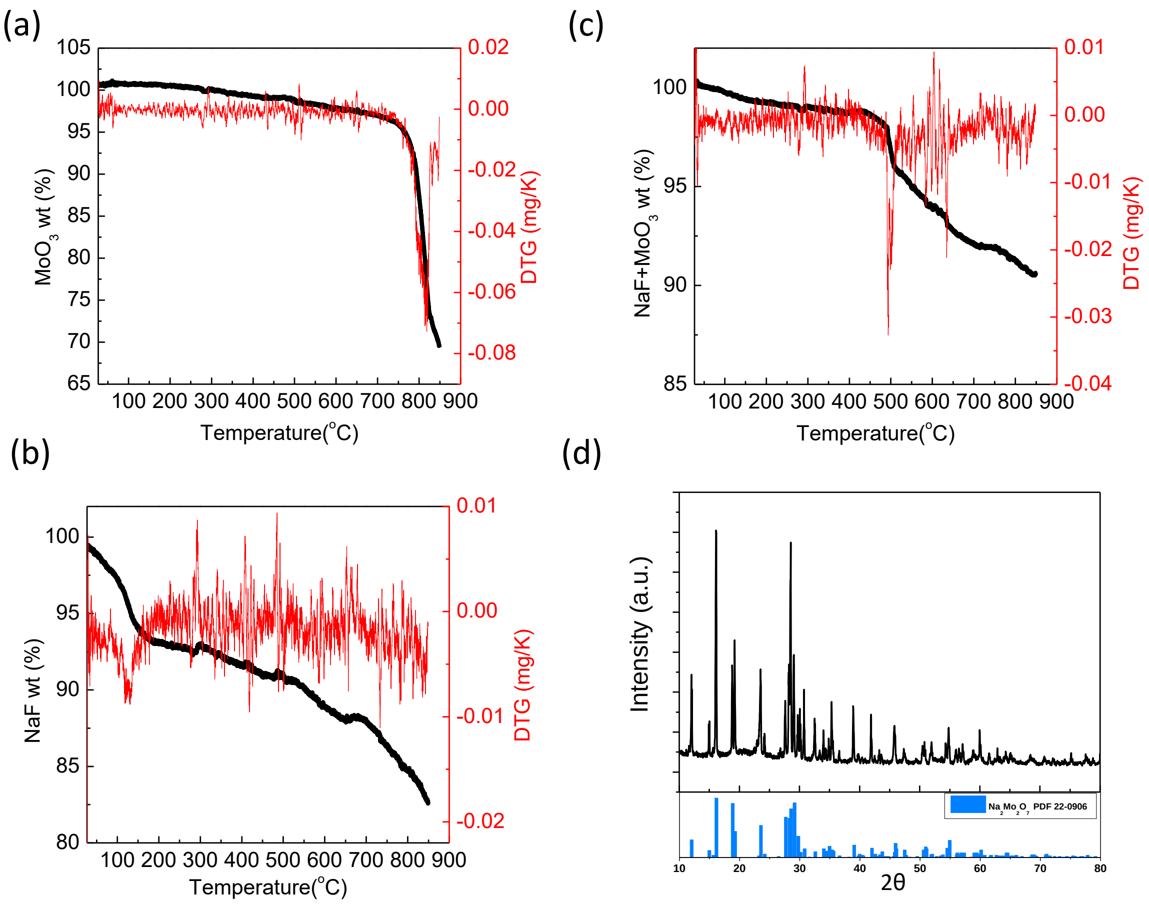

Supplementary Figure 3. Thermogravimetric analysis. (TGA) of the reaction between NaF and MoO_3. TGA data of (a) MoO_3 (b) NaF (c) NaF+MoO_3. The decrease in mass of NaF sample at temperature lower than 200 °C is related to the evaporation of absorbed water vapor. The sharp exothermal peak at about 500 °C in (c) indicates the reaction between NaF and MoO_3, which forms $Na_2Mo_2O_7$ and MoO_2F_2. MoO_2F_2 is in gas phase at the reaction temperature and is pumped out from the system, causing the decrease in total mass. The remaining liquid phase $Na_2Mo_2O_7$ has a low vapor pressure, indicated by the little weight loss of $Na_2Mo_2O_7$ liquid even at temperature as high as 850 °C, which is favorable for SCVLS process. The remainder powder after cooling was examined by X-ray diffraction and the result is shown in (d). All peaks fit well to the predicted $Na_2Mo_2O_7$ diffraction peaks.

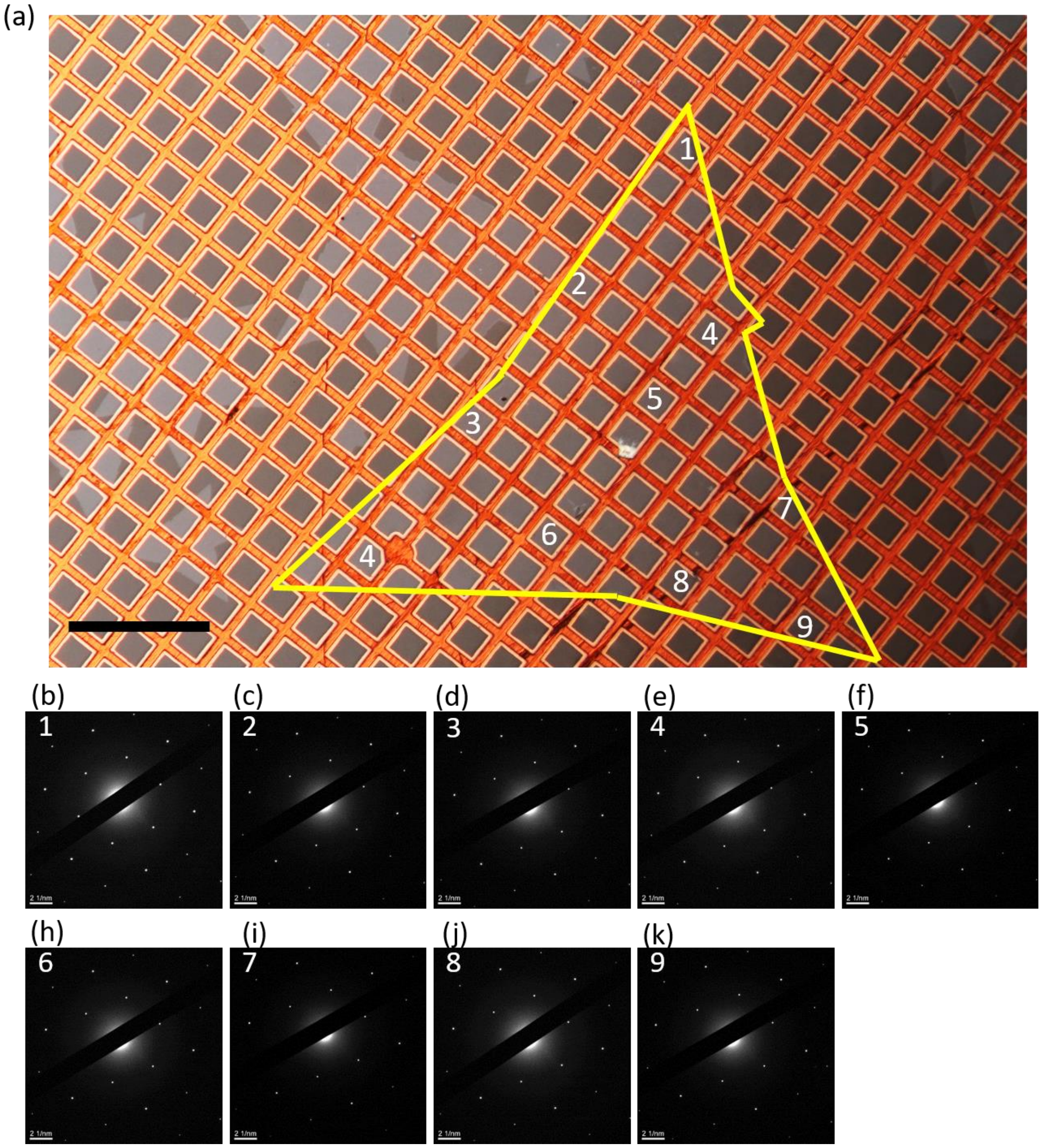

Supplementary Figure 4. Single-crystal property of large, non-perfectly-triangular MoS_2 flakes. (a) Optical microscopy image of the transferred MoS_2 flakes on a 400 mesh TEM grid. Scale bar is 200 µm. (b)-(k) The diffraction patterns taken in the grids labeled 1 to 9, respectively. The diffraction patterns taken in different areas inside a single grain are identical, proving that the large grains grown by this method are single crystals, even though they do not possess a perfect triangular shape.

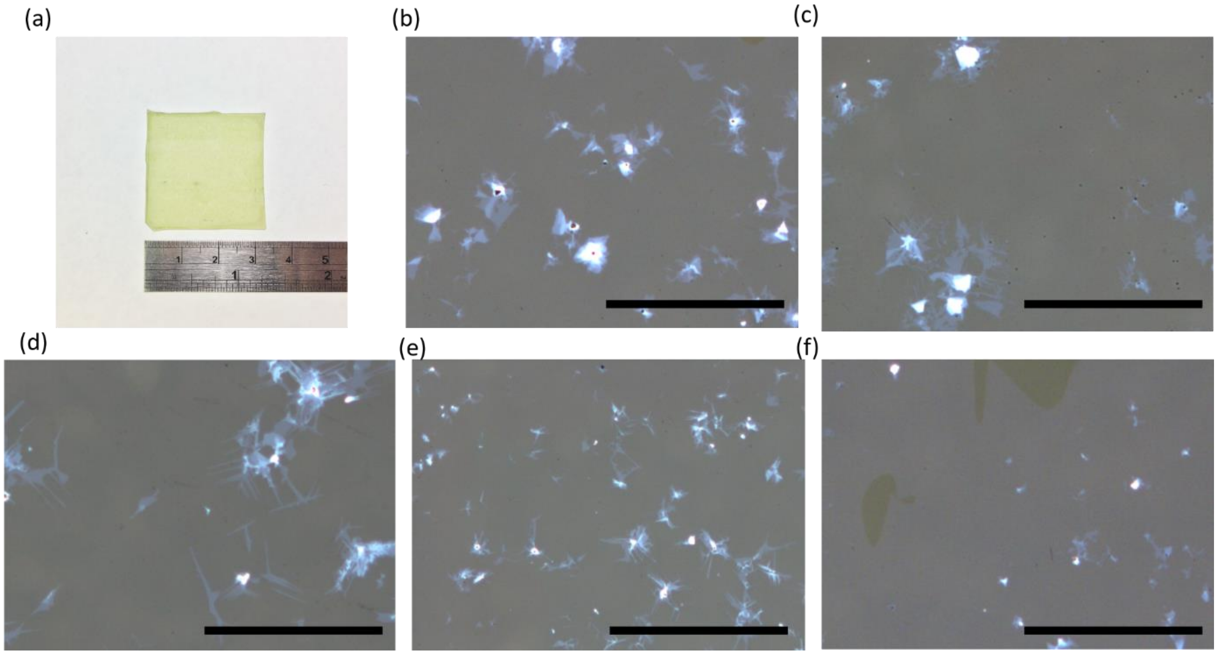

Supplementary Figure 5. Full-coverage MoS_2 film grown on top of a 3 × 3 cm^2 c-plane sapphire substrate. (a) Photo image of as-grown full-coverage film. (b)-(f) Optical images of the continuous film. Scale bars are 250 µm. (f) is taken closer to the edge of the sample to show the contrast of the area covered with MoS_2 and areas that are not. The SCVLS method can extend to even larger substrate as long as the substrate can fit in the plasma-enhanced atomic layer deposition chamber and the CVD tube.

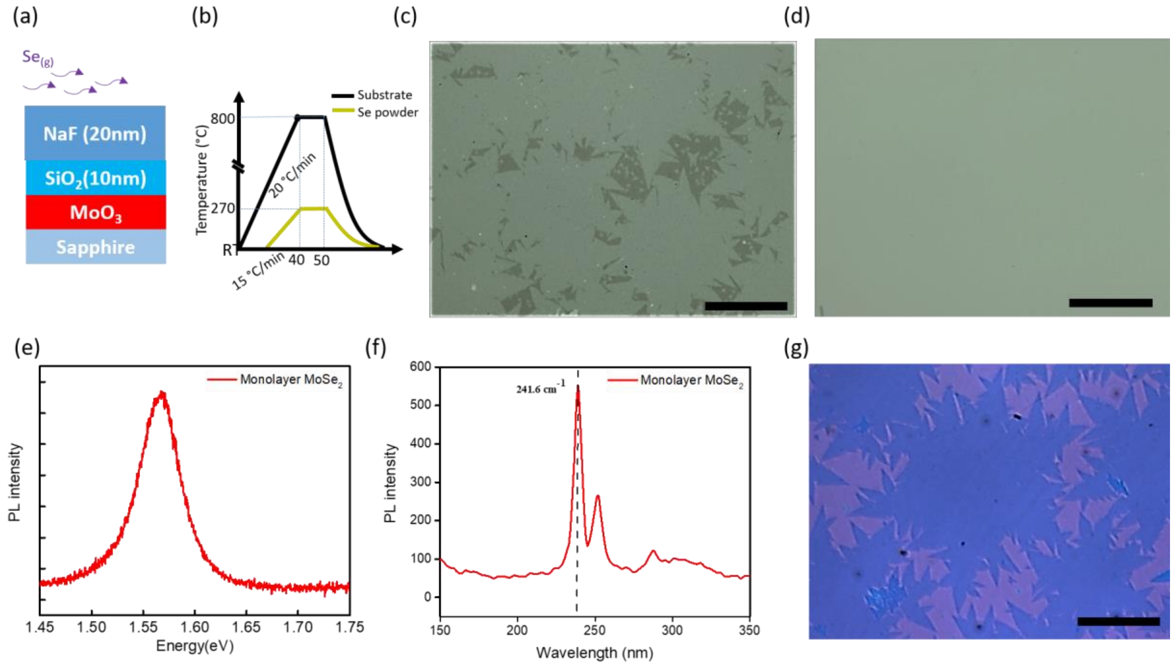

Supplementary Figure 6. SCVLS growth of $MoSe_2$. (a) Structure of precursor. (b) Temperature ramping profile of the process. (c) and (d) are the optical images of as-grown $MoSe_2$ with 6-nm and 8-nm MoO_3 precursors. The scale bars are 150 μ m. (e) Photoluminescence and (f) Raman spectra of as-grown $MoSe_2$. (g) Transferred $MoSe_2$ on the silicon substrate with a 300 nm thermal oxide layer. The scale bar is 150 μm.

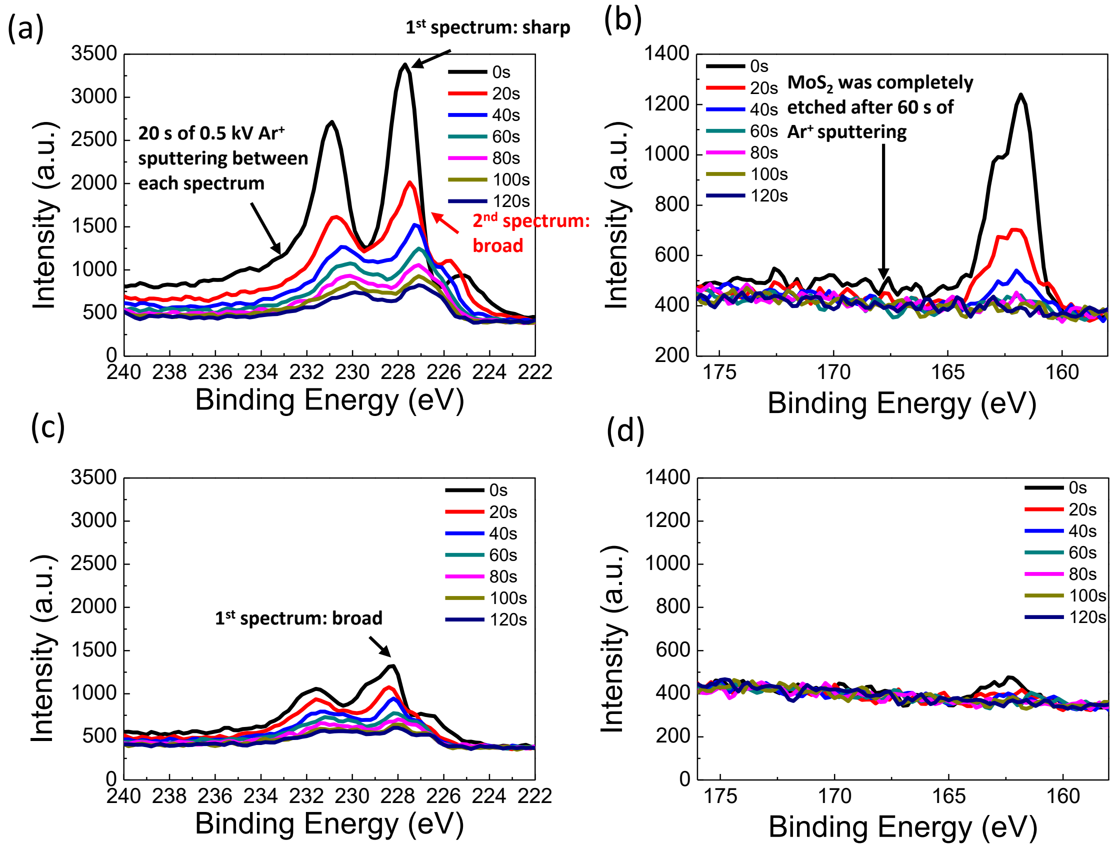

Supplementary Figure 7. XPS depth profile of as-grown sample. (a) Mo-3d (b) S-2p signal of sites that are cover by MoS_2 and (c) Mo-3d (d) S-2p signal of sites that are not covered by MoS_2. At sites that are covered by MoS_2 layer (a and b), the signal consists of top crystalline MoS_2 (sharp) and quenched solid solution (broad). After Ar plasma etching, the Mo-3d spectrum started to broaden because of the removal of top MoS_2 layer. The Mo-3d and S-2p spectra of the uncovered sites (c,d) are broad at the beginning and has much weaker intensity. The decrease in sulfur content with depth at both sites is an indication of the downward diffusion of sulfur.

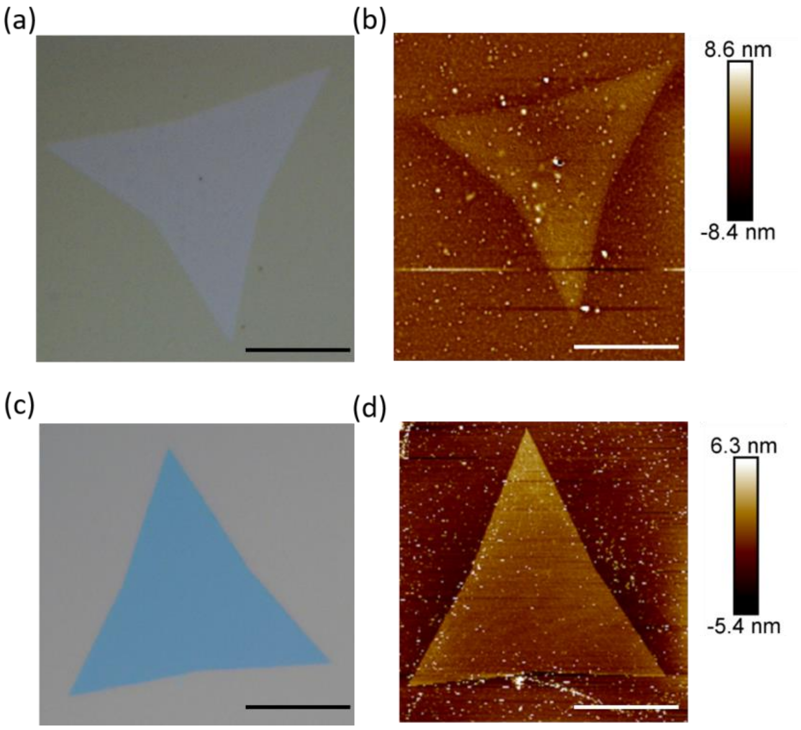

Supplementary Figure 8. AFM analysis of SCVLS MoS_2 (a) Optical and (b) AFM images of the as-grown MoS_2. (c) Optical and AFM images of the transferred MoS_2 on silica. All scale bars in the images are 20 µm. The average roughness of (b) and (d) are 0.29 and 0.19 nm, respectively.

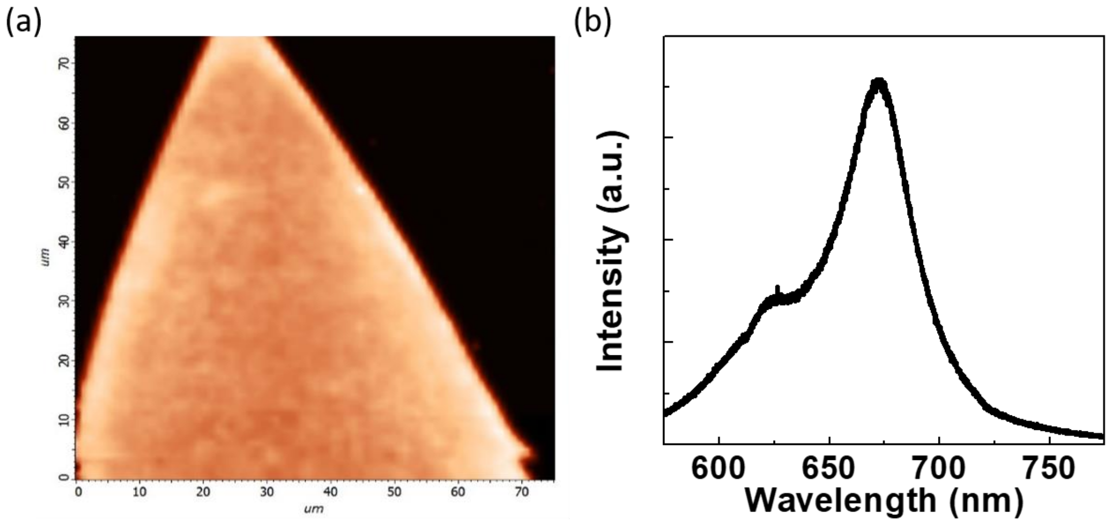

Supplementary Figure 9. Optical properties of the as-grown MoS_2. (a) Photoluminescence mapping image of the as-grown MoS_2 on sapphire. (b) Photoluminescence spectrum.

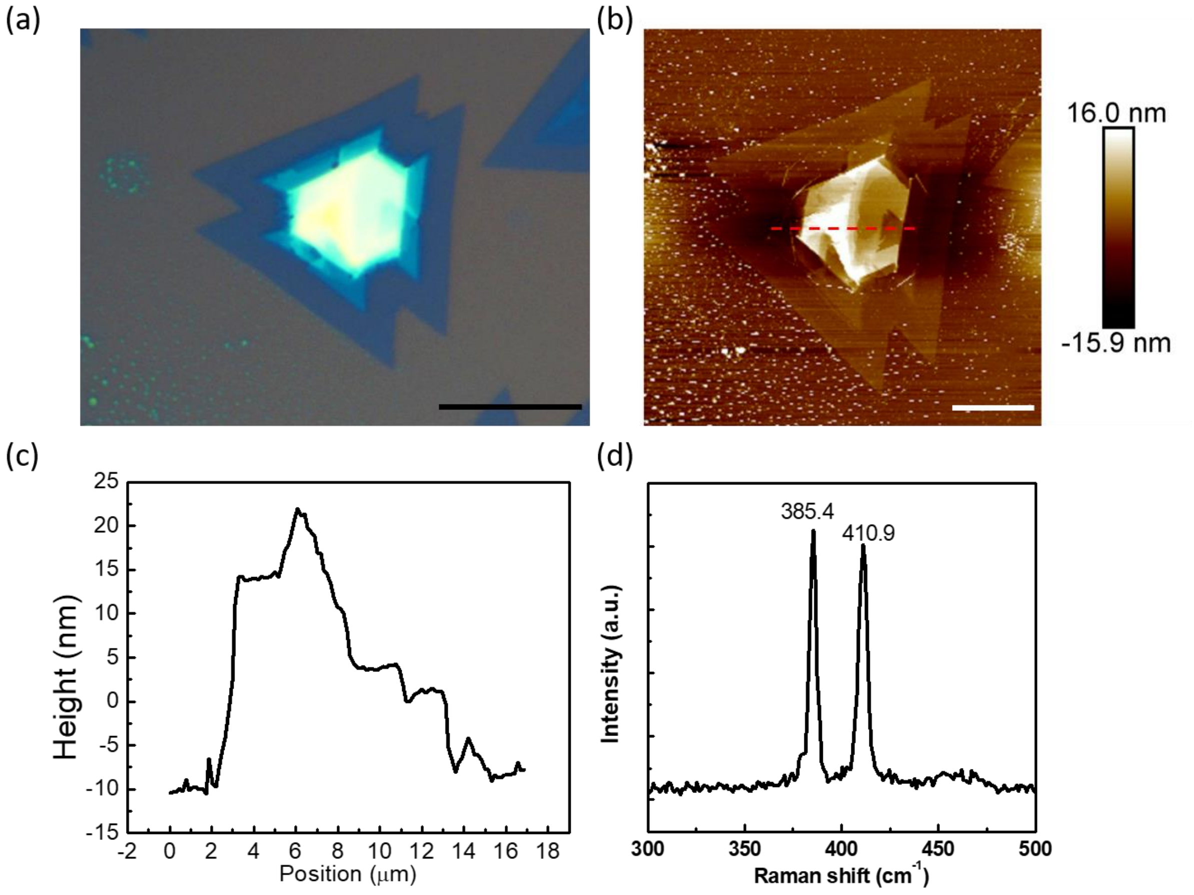

Supplementary Figure 10. Characterizations of the white dots (flake) on a monolayer grain. (a) Optical image of the white dot. The scale bar is 20 μm. (b) AFM image of the white dots. The scale bar is 8 μm. Height profiles is shown in (c). (d) Raman spectrum of the white dots. We speculate these white dots come from the liquid precursor that overflowed to the top during the growth of MoS_2 and eventually sulfurized into MoS_2 flake on top of the monolayer.

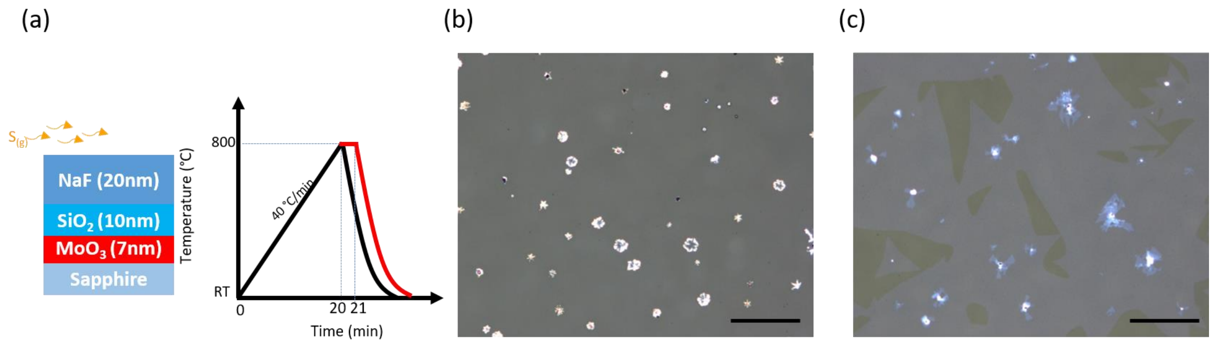

Supplementary Figure 11. Growth rate of SCVLS. (a) Two different growth periods (0 and 1 minute) are used here. The as-grown optical images are shown in (b) and (c). The scale bars in b and c are 200µm. For 0 min growth time, there is no clear MoS_2 (only with non-fully sulfurized $Na_2Mo_2O_7$ on or inside the NaF matrix). For 1-minute growth, the average MoS_2 grain size is 370 µm. The growth rate is calculated as 370 or 214 µm /min (calculated by the edge length or the length from geometry center to tip), which is faster than most reported CVD methods.

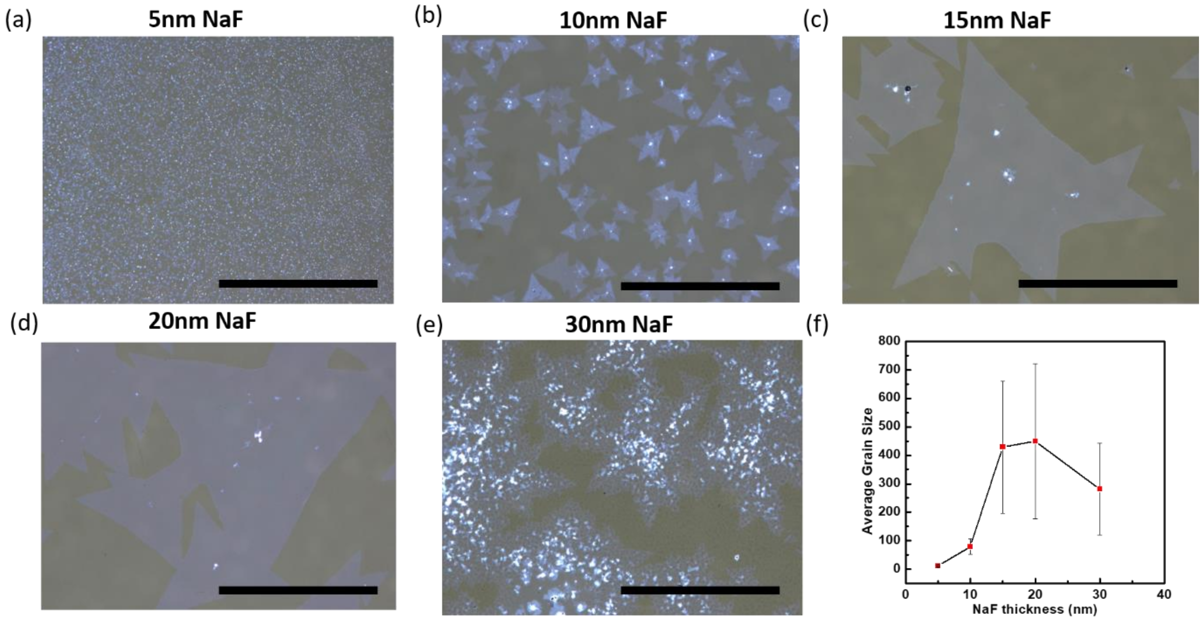

Supplementary Figure 12. NaF thickness effect. Optical images of SCVLS MoS_2 with a (a) 5 nm, (b) 10 nm, (c) 15 nm, (d) 20 nm, and (e) 30 nm NaF layer. The thickness of MoO_3 and SiO_2 are 5 and 10 nm, respectively. Scale bars are 500 µm in (a)-(e). The average grain size of MoS_2 grown with different NaF thickness is shown in (f). The grain size of MoS_2 increases firstly as NaF gets thicker because the thicker NaF reduces the liquid droplet density during the growth stage. However, if the NaF is too thick, it is difficult for liquid to go up to surface. With a lot of liquid at the bottom, the whole structure become very unstable, so the morphology of MoS_2 become very non-uniform as shown in (e). Here, 15~20 nm is found to be the best thickness for growing large MoS_2 crystals.

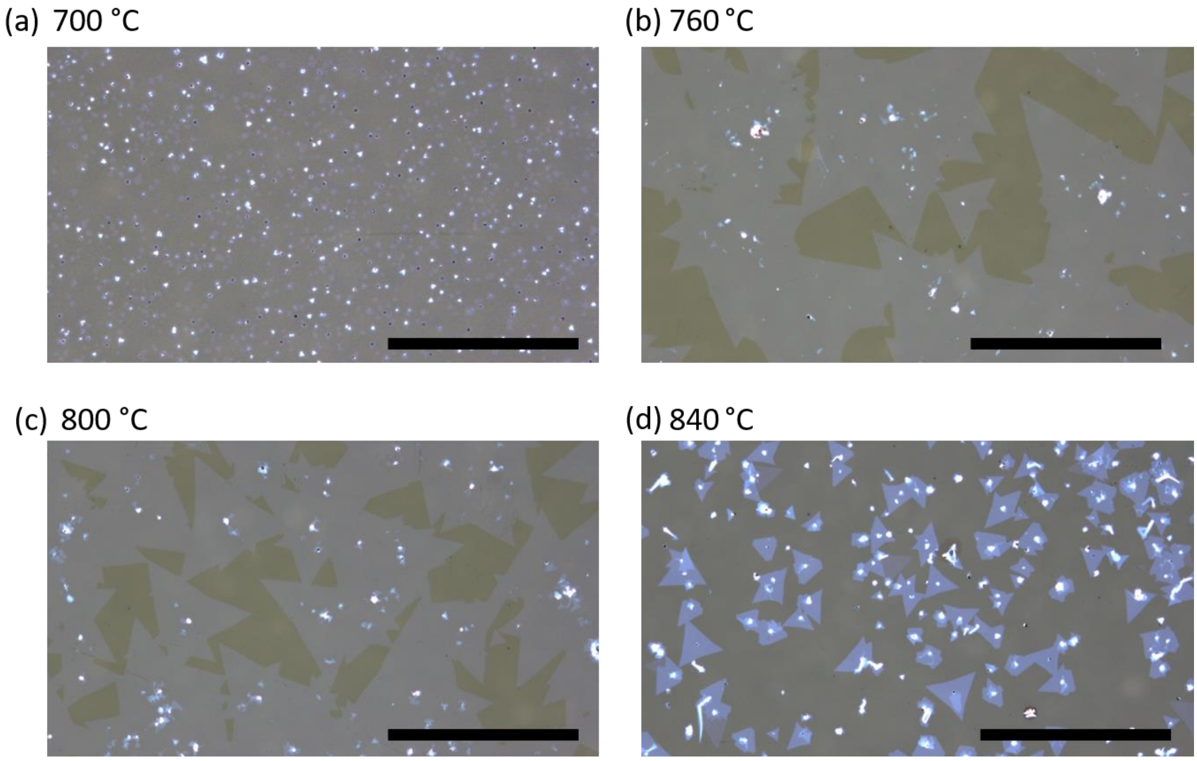

Supplementary Figure 13. Temperature effect on SCVLS growth. Optical images of SCVLS MoS_2 grown at (a) 700 ℃ (b) 760 ℃ (c) 800 ℃ (d) 840 ℃. As described in the manuscript, the SCVLS reaction needs enough MoO_3 to break the SiO_2 diffusion membranes and react with NaF. Therefore, for low temperature (700 ℃) as shown in (a), the lack of enough liquid on top results in small and low coverage MoS_2 crystals. If the temperature is too high (840 ℃ as shown in d), the MoO_3 vapor pressure is too high and results in too much liquid on the top surface before the sulfurization process so a smaller bilayer structure is dominated at this condition. The best condition of uniform monolayer is between 760~800 ℃ (b and c) range, which also depends on the amount of MoO_3 and SiO_2 that are used. The thickness of MoO_3, SiO_2, and NaF are 5, 10, and 20 nm, respectively. Scale bars are 500 μm.

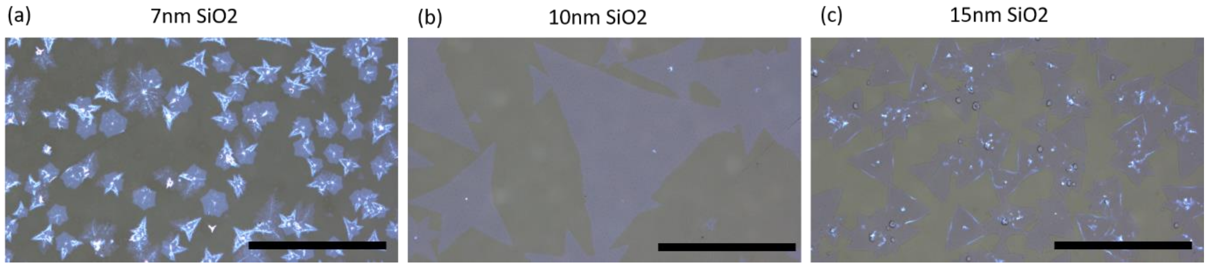

Supplementary Figure 14. SiO_2 thickness effect. The MoS_2 grain grown using precursors with (a) 7 nm (b) 10 nm (c) 15 nm-thick SiO_2 diffusion membrane. The size of MoS_2 grain increases with SiO_2 thickness firstly and then deceases with SiO_2 thickness. When the thickness of SiO_2 is not enough, the sputtered SiO_2 layer cannot form a fully covered film, letting the MoO_3 and NaF film to be in contact and react at a lower temperature. The excess liquid phase product, $Na_2Mo_2O_7$, will make the surface instable and will make the as-formed MoS_2 crumble and also favor multilayer growth. With suitable SiO_2 thickness, the MoS_2 flake can grow into large flake, as in (b). When the SiO_2 layer is too thick (15 nm), the vapor pressure of MoO_3 needed to break the SiO_2 layer is higher. The vertical driving force of growth is weaker, so the average grain size becomes smaller than 10 nm SiO_2. The unstable MoO_3 and $Na_2Mo_2O_7$ at the bottom result in a worse morphology. The thickness of MoO_3 and NaF are 5 and 20 nm, respectively. Scale bars are 500 µm.

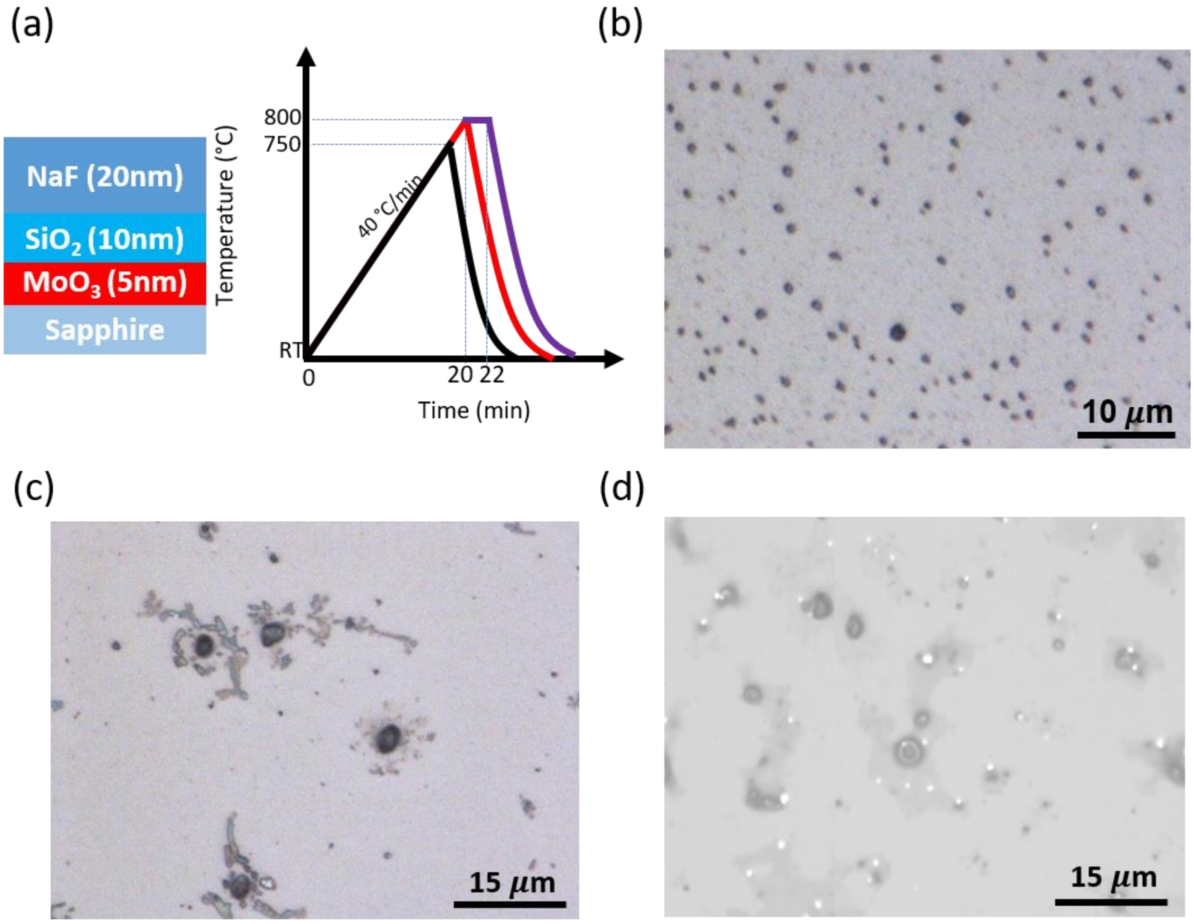

Supplementary Figure 15. Dynamic control of eutectic reaction (without sulfurization). (a) Different heating time and temperature are used to control the size of droplets. (b), (c) and (d) are the as-grown optical images correspond to the blue, red and purple ramping curves shown in (a). The $Na_2Mo_2O_7$ particles started appearing at 750 ^{0}C (about 0.2-1 um size shown in (b)) and the size of droplets became larger as the temperature increases to 800 ^{0}C (about 1-10 um size shown in (c)), which indicated the liquid gradually rose up on the NaF surface. When we maintained at 800 ^{0}C for additional 2 minutes, the particle became larger and started wetting the surface (the wetting area is about 100-400 um^2 as shown in (d)), explaining why we can grow bilayer structure by delaying the timing of sulfurization as described in the manuscript (Figure 4).

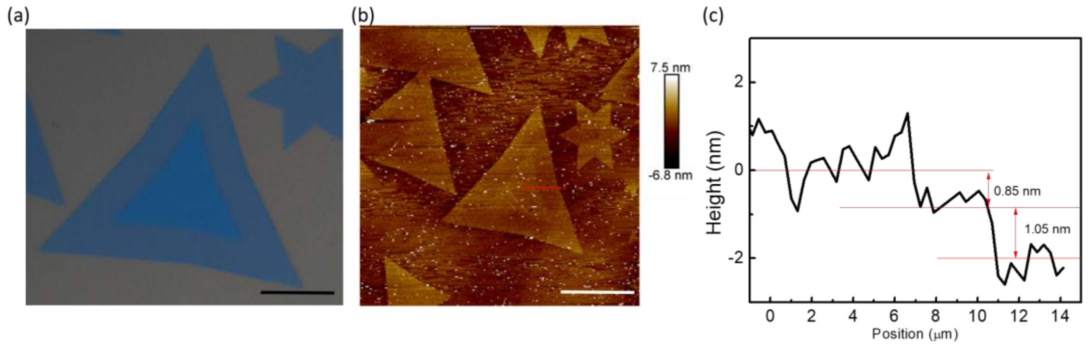

Supplementary Figure 16 AFM measurement of MoS_2 on a silicon substrate with a 300-nm thermal oxide. (a) Optical image of a bilayer flake. The scale bar is 10 μm (b) AFM image of the bilayer MoS_2 flake. The scale bar is 20 μm. (c) Height profile of the dash line in (b).

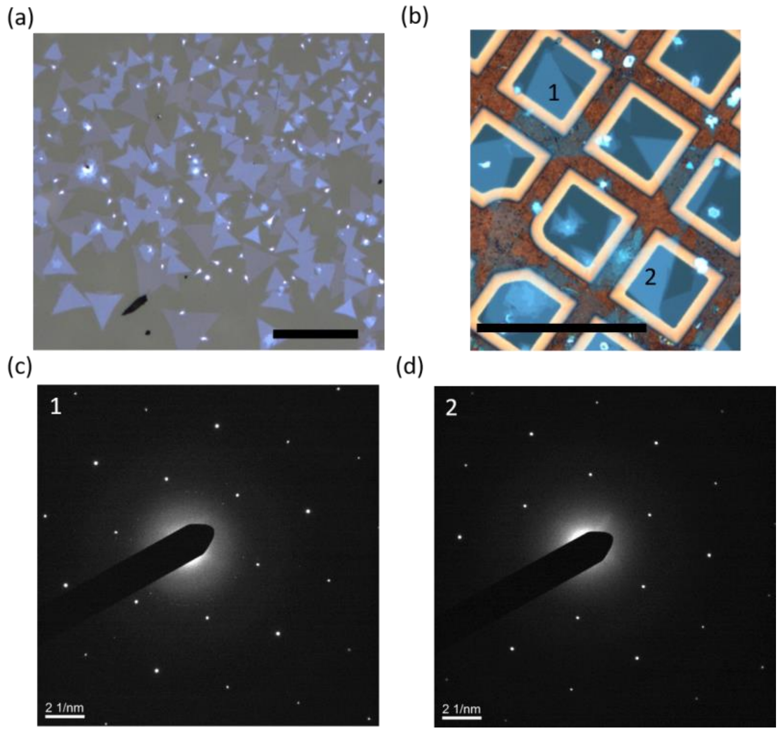

Supplementary Figure 17. Crystal structure of multilayer MoS_2 grown by SCVLS. Optical image of (a) as-grown and (b) transferred multilayer MoS_2. Judging from optical contrast, label 1 is a bilayer grain and label 2 is a trilayer grain. Scale bars are 200 μm in (a) and 100 μm in (b). (c) Diffraction pattern of grain 1. (d) Diffraction pattern of grain 2. The identical diffraction patterns to the monolayer MoS_2 crystals indicate they have a 2H stacking structure.

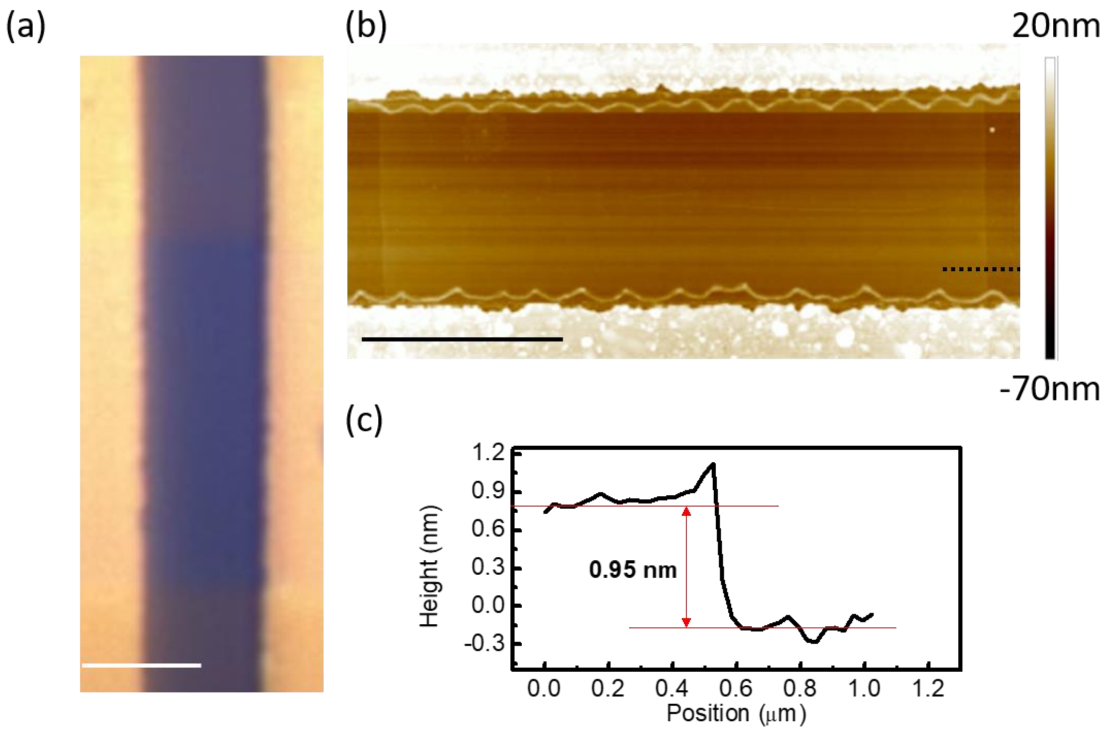

Supplementary Figure 18. AFM measurement of FET devices. (a) Optical and (b) AFM images of the monolayer MoS_2 device. The jagged edges of electrodes are due to the metal liftoff process. The scale bars in (a) and (b) are 5 μm. (c) Height profile of the monolayer MoS_2.

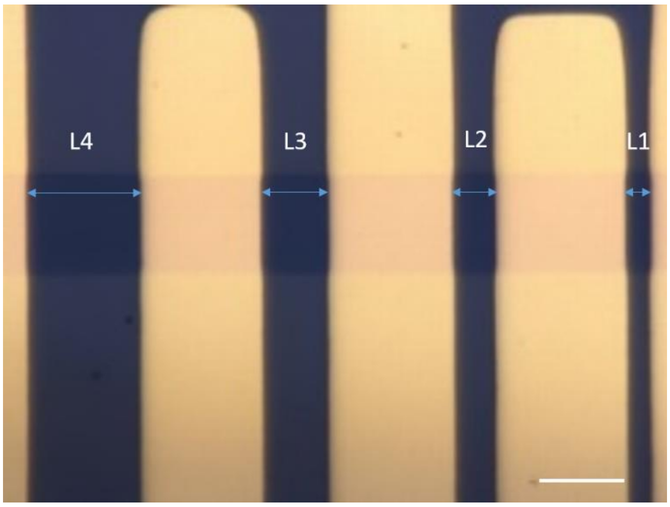

Supplementary Figure 19. FET devices with different channel lengths. The channel lengths of L1-L4 are 1.48, 2.38, 3.90 and 6.60 μm, respectively. The scale bar is 5 μm.

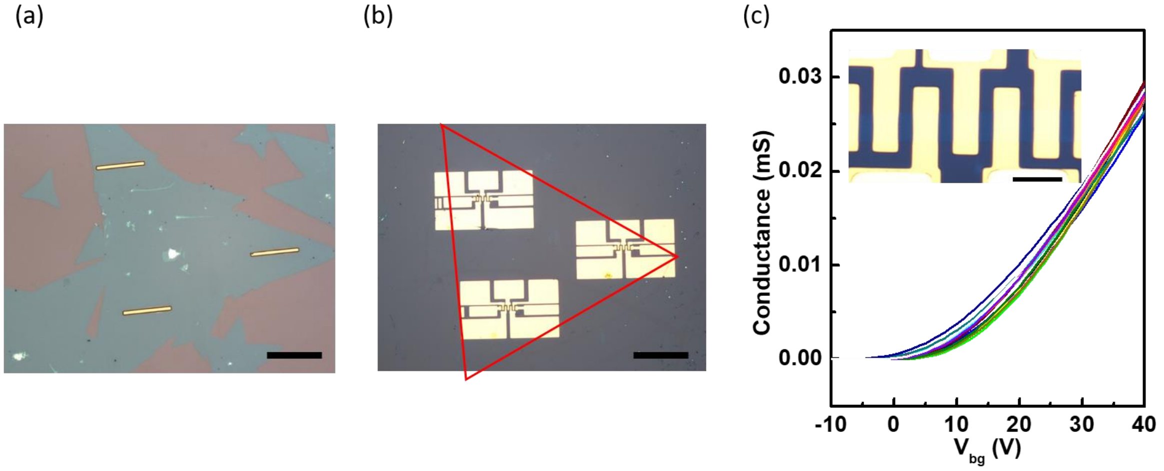

Supplementary Figure 20. Transport properties of FETs in a 1 mm monolayer MoS_2 crystal. (a) Optical image of a 1-mm MoS_2 crystal on SiO_2. The three yellow stripes are the photoresist for patterning MoS_2. Scale bar is 200 µm. (b) Optical image of 18 FET devices fabricated using the three MoS_2 stripes as defined in (a). Each ribbon had six identical devices as shown in the inset of (c). (c) Gate-dependent conductance of 18 FET devices in a 1-mm MoS_2 crystal. All devices exhibited similar field-effect mobility (33 ± 3 cm^2 V^{-1} S^{-1}). The inset is the optical image of devices. Scale bar is 25 µm.

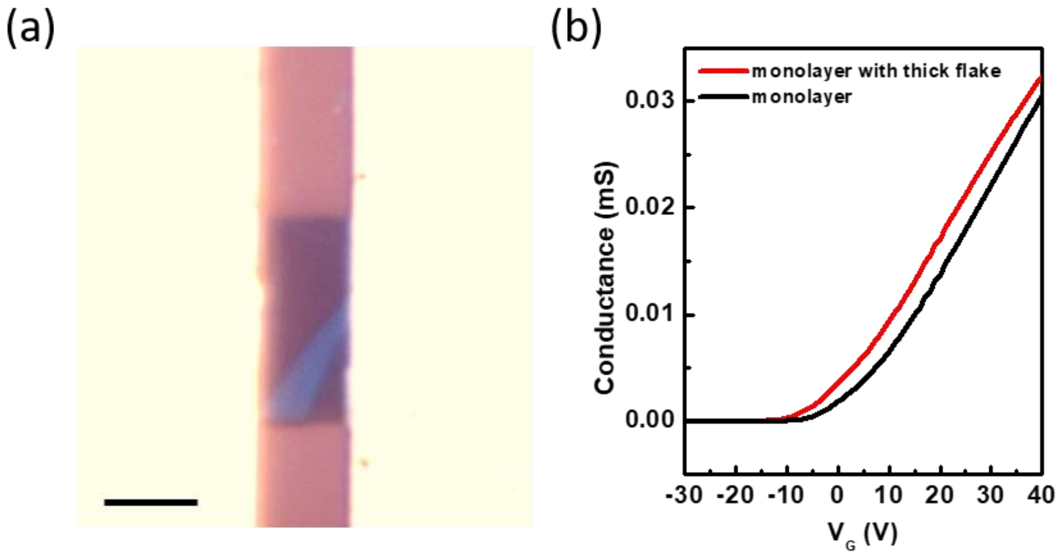

Supplementary Figure 21. Monolayer device with a top flake. (a) Optical image of a monolyaer device with a MoS_2 flake on top of it. The sclae bar is 5 μm. (b) Transport properties of the device with pure monolayer and monolayer with a top flake.

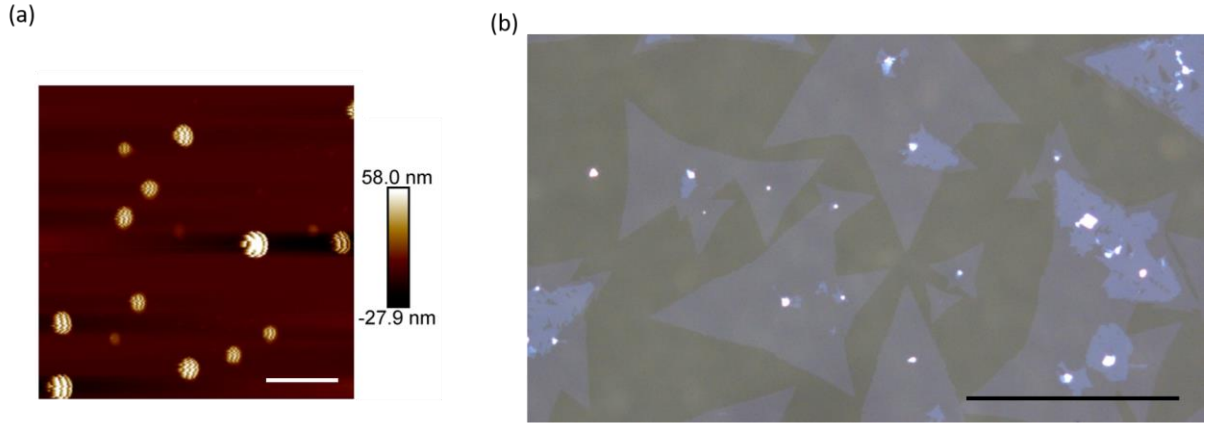

Supplementary Figure 22. SCVLS method with evaporated MoO_3 precursors. (a) The morphology of evaporated 2 nm MoO_3 film. Some island-like MoO_3 clusters is observed in the evaporated film. (b) 500 um grain size MoS_2 can still be grown using evaporated MoO_3. However, the worse morphology of MoS_2 grains may result from the worse morphology of evaporated MoO_3. Scale bar in (a) is 2 μm and (b) is 500 μm.

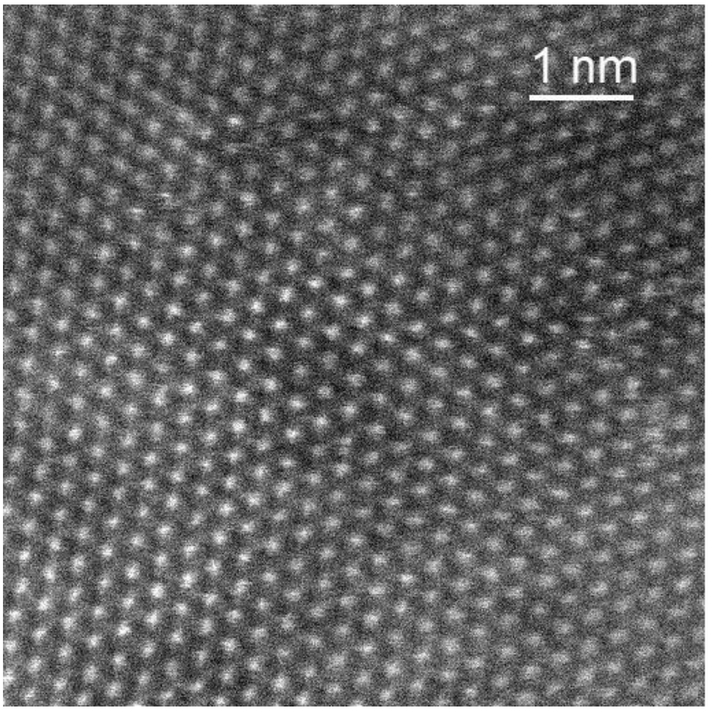

Supplementary Figure 23. Atomic-resolution high-angle annular dark-field scanning transmission electron microscopy (HAADF-STEM) image for the SCVLS MoS_2. The hexagonal lattice shown in HAADF-STEM image is the basal plane (0001) of MoS_2. Because the image intensity in HAADF-STEM mode is proportional to $Z^{1.7}$ (so-called Z-contrast image), where Z is the atomic number, the contrast of the Mo atoms is much higher than that of the S atom. The measured lattice constant of the SCVLS MoS_2 is 0.306 nm, which is close to the theoretic value.

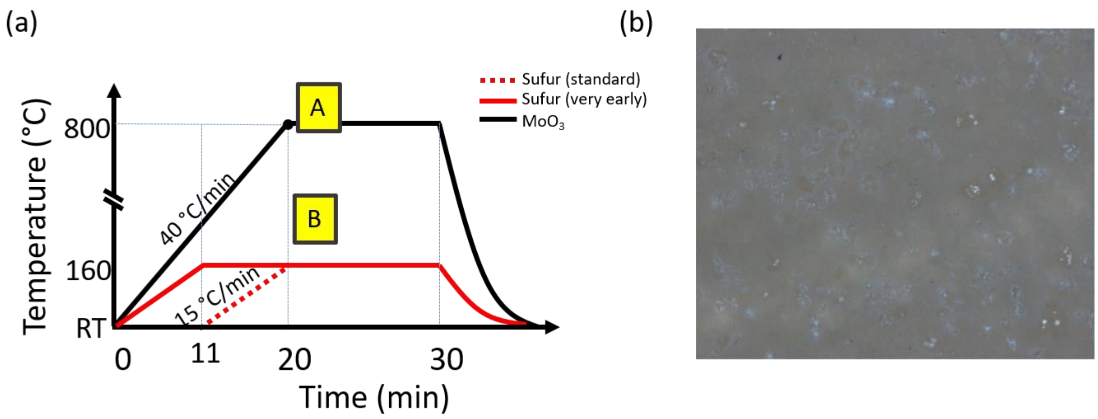

Supplementary Figure 24. Growth with an early sulfurization condition. (a) Temperature ramping profile of the growth with a very early sulfurization (b) Optical image of the as-grown product with a very early sulfurization condition.
